# Costs of diabetes complications: hospital-based care and absence from work for 392,200 people with type 2 diabetes and matched control participants in Sweden

**DOI:** 10.1007/s00125-020-05277-3

**Published:** 2020-09-24

**Authors:** Emelie Andersson, Sofie Persson, Nino Hallén, Åsa Ericsson, Desirée Thielke, Peter Lindgren, Katarina Steen Carlsson, Johan Jendle

**Affiliations:** 1 The Swedish Institute for Health Economics, Lund, Sweden; 2 Health Economics Unit, Department of Clinical Sciences, Malmö, Lund University, Lund, Sweden; 3 Novo Nordisk A/S, Copenhagen, Denmark; 4 Novo Nordisk Scandinavia, Malmö, Sweden; 5 Department of Learning, Informatics, Management and Ethics, Karolinska Institutet, Stockholm, Sweden; 6 Diabetes, Endocrinology and Metabolism Research Centre, Institute of Medical Sciences, Örebro University, Örebro, Sweden

**Keywords:** Costs and cost analysis, Diabetes complications, Diabetes mellitus, type 2, Hospital costs, Insurance, disability, Sick leave

## Abstract

**Aims/hypothesis:**

The risk of complications and medical consequences of type 2 diabetes are well known. Hospital costs have been identified as a key driver of total costs in studies of the economic burden of type 2 diabetes. Less evidence has been generated on the impact of individual diabetic complications on the overall societal burden. The objective of this study was to analyse costs of hospital-based healthcare (inpatient and outpatient care) and work absence related to individual macrovascular and microvascular complications of type 2 diabetes in Sweden in 2016.

**Methods:**

Data for 2016 were retrieved from a Swedish national retrospective observational database cross-linking individual-level data for 1997–2016. The database contained information from population-based health, social insurance and socioeconomic registers for 392,200 people with type 2 diabetes and matched control participants (5:1). Presence of type 2 diabetes and of diabetes complications were derived using all years, 1997–2016. Costs of hospital-based care and of absence from work due to diabetes complications were estimated for the year 2016. Regression analysis was used for comparison with control participants to attribute absence from work to individual complications, and to account for joint presence of complications.

**Results:**

Use of hospital care for complications was higher in type 2 diabetes compared with control participants in 2016: 26% vs 12% had ≥1 hospital contact; there were 86,104 vs 24,608 outpatient visits per 100,000 people; and there were 9894 vs 2546 inpatient admissions per 100,000 people (all *p* < 0.001). The corresponding total costs of hospital-based care for complications were €919 vs €232 per person (*p* < 0.001), and 74.7% of costs were then directly attributed to diabetes (€687 per person). Regression analyses distributed the costs of days absent from work across diabetes complications per se, basic type 2 diabetes effect and unattributed causes. Diabetes complications amounted to €1317 per person in 2016, accounting for possible complex interactions (25% of total costs of days absent). Key drivers of costs were the macrovascular complications angina pectoris, heart failure and stroke; and the microvascular complications eye diseases, including retinopathy, kidney disease and neuropathy. Early mortality in working ages cost an additional €579 per person and medications used in risk-factor treatment amounted to €418 per person.

**Conclusions/interpretation:**

The economic burden of complications in type 2 diabetes is substantial. Costs of absence from work in this study were found to be greater than of hospital-based care, highlighting the need for considering treatment consequences in a societal perspective in research and policy.

Graphical abstract
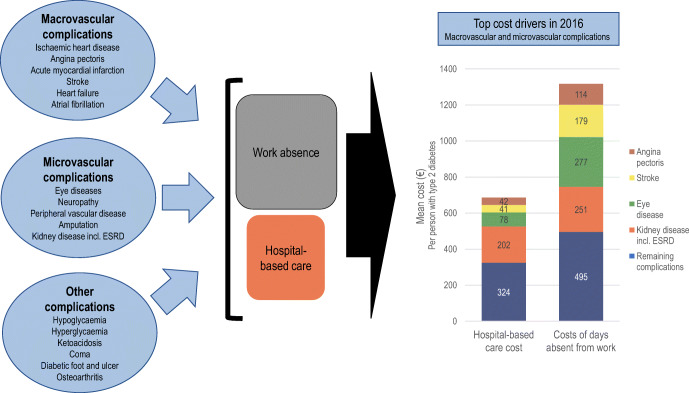

**Electronic supplementary material:**

The online version of this article (10.1007/s00125-020-05277-3) contains peer-reviewed but unedited supplementary material, which is available to authorised users.

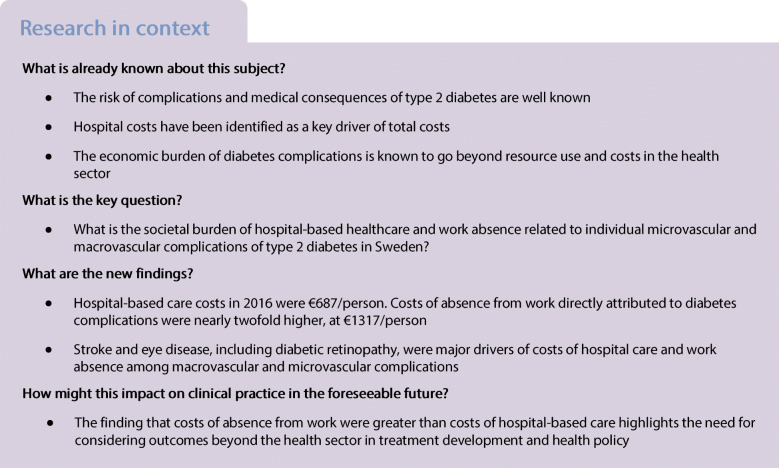



## Introduction

Type 2 diabetes is a prevalent and costly chronic disease associated with several health complications. Worldwide, the prevalence of diabetes has increased over the last decades [1] and, in 2019, there were about 463 million adults with diabetes according to the International Diabetes Federation [2]. Rising health expenditure in absolute values and as percentage of gross national product, as shown in, for instance, Organisation for Economic Cooperation and Development (OECD) health data [3], is a concern for decision makers in national health systems as well as insurance-based systems.

The economic burden of diabetes complications is known to go beyond resource use and costs in the health sector. Previous studies on the cost burden of type 2 diabetes have demonstrated that costs from reduced productivity (including e.g. work days absent, reduced labour force participation, mortality) constitute a substantial part of the total cost burden, as shown by, for instance, the most recent updates on economic costs of diabetes in the USA from 2017 and in the UK from 2012 [4–6]. Less evidence has been generated on the economic impact of individual diabetic complications and little is known about which complications are the key drivers of the overall societal burden. The identification of key cost drivers among complications informs research and policy of where further actions and tools are needed. Such data support clinicians, employers, organisations and society with facts on the value of preventing and postponing the incidence of diabetes complications and the extent of the economic burden falling on the individual where insurance coverage is lacking or inadequate.

Studies on the cost of illness combine data from a mix of sources, often using aggregate costs where a part of the costs is attributed to the specific disease. Complications related to type 2 diabetes are conditions that may also occur in the general population, such as cardiovascular diseases. Hence, analyses of the costs of type 2 diabetes usually report the total costs incurred by people with diabetes and the cost attributed to diabetes when applying top-down estimation strategies. For example, the ADA's report on the economic costs of diabetes in 2017 attributed 57% of the hospital inpatient care costs incurred by people with diabetes to diabetes per se (US$70 billion of US$123 billion) [4]. Costs of disease may also be derived from individual-level data with population coverage, preferably holding longitudinal information from multiple administrative registers. A Danish study of number of days absent from work due to diabetes-related complications showed that several of the investigated complications led to more than 3 months of work absence for the average person with diabetes [6].

Recent years have seen an increase in studies using diagnostic codes and administrative registers to identify people with diabetes and conduct epidemiological, clinical and economic research. Some examples include studies on total costs of care for people with diabetes [7–10], on prevalence and incidence of diabetes [11, 12] and on types of glucose-lowering medication and clinical outcomes [13]. Similar topics have also been addressed using patient cohorts registered in quality registers or incidence registers and using outcome data from national health registers in Sweden [14–21].

Sweden has a long tradition of reporting population-based information into registers, and data can be record-linked between registers for research using the Swedish personal identification number. Register-based definitions of diabetes may rely on diabetes diagnosis solely [12] or use a combination of observed healthcare contacts with diabetes diagnosis and filled prescriptions of different types of glucose-lowering medications [8, 13, 22]. This study used the latter strategy with the purpose of estimating costs of hospital (inpatient and outpatient) care and costs of absence from work related to individual macrovascular and microvascular complications of type 2 diabetes in Sweden. Using cross-linked microdata, matched control participants and straight-forward empirical strategies, this study addresses previously identified research gaps in the economic costs of type 2 diabetes and the contributions of individual diabetes complications. It also provides new insights into the balance of costs between the healthcare sector and other sectors.

## Methods

### Data

The analysis of the cost burden of type 2 diabetes complications in 2016 used data from a retrospective observational study database [22]. The database cross-links 20 years of individual-level data, 1997–2016, from three national authorities in Sweden: the National Board of Health and Welfare (NBHW), Försäkringskassan (the Swedish Social Insurance Agency) and Statistics Sweden. The NBHW identified people with diabetes and provided data from the National Patient Register (NPR) on outpatient and inpatient hospital healthcare [23], and from the National Prescribed Drugs Register (NPDR) on filled prescriptions of selected drugs [24]. Further register description is available in electronic supplementary material (ESM) Table 1. Demographic and socioeconomic background characteristics were obtained from Statistics Sweden from the Register of the Total Population and the LISA database (longitudinal integrated database for health insurance and labour market studies [25]). Försäkringskassan supplied data on days absent from work from the MiDAS database (Micro-Data for the Analysis of Social Insurance) covering schemes of sickness and rehabilitation benefits, and sickness and activity compensation [26]. This study calculated costs of complications in the year 2016 based on use of hospital care and absence from work in 2016. To identify the population with type 2 diabetes and presence of diabetes complications, we used the full longitudinal database (1997–2016).

People with diabetes of labour market active ages (16–70 years) were selected for the database if they, at any point during 1997–2016, fulfilled at least one of the following criteria:

1. At least one healthcare visit or inpatient stay with diabetes as main or secondary diagnosis by the International Classification of Diseases version 10 (ICD 10; http://apps.who.int/classifications/icd10/browse/2016/en) codes for type 1 diabetes (E10), type 2 diabetes (E11) or unspecified diabetes (E14) in any year in the NPR from 1997 to 2016.
2. At least two dispensed prescriptions of glucose-lowering medication (Anatomic Therapeutic Chemical (ATC) Classification System codes, A10) with dispense dates not more than 6 months apart in the NPDR, from 1 July 2005 to 31 December 2017.

Women treated with glucose-lowering medication with a coinciding registration of pregnancy were not included in the study population but were eligible again after 2 years. An index date was defined as the first documented occurrence from 1997 and onwards in the NPR, or the first documentation in the NPDR from 1 July 2005 and onwards, whichever occurred first.

For each person with diabetes, Statistics Sweden matched control participants from the general population (5:1) using exact year of birth, sex and region of residence in the index year. This enabled incremental analysis of costs of hospital care and absence from work for people with diabetes compared with control participants. The design allowed control participants to switch to the diabetes group at a later stage if they then fulfilled inclusion criteria for diabetes. Further information on the data sources, retrieval process and control selection can be found in the ESM (pages 2–6).

The study database includes background data (demographic and socioeconomic), exposure data (diabetes complications) and outcome data (healthcare use, filled prescriptions, mortality and days absent from work).

### Empirical strategies

#### Selecting the type 2 diabetes population

The empirical definition of type 2 diabetes was similar to previous registry-based diabetes studies [7, 8, 13]. From the study database we omitted people with type 1 diabetes defined as having inpatient and/or outpatient care registered with type 1 diabetes (ICD E10), but no registration of type 2 diabetes (E11) or unspecified diabetes (E14), as main or supplementary diagnosis in the NPR during the study period. All other cases were included as type 2 diabetes (see the ESM for more information on selection process).

#### Defining diabetes complications

Complications were defined using ICD codes and procedure codes (Classification of Healthcare Interventions [Klassifikation av. vårdåtgärder (KVÅ); NBHW], which holds surgical and non-surgical interventions including diagnostic procedures in line with previous studies, as referenced in ESM Table 2). The incidence and prevalence rates of complications were identified through registrations of main diagnosis in hospital-based care. The empirical strategy was not expected to capture all early stages of complications, non-emergency needs and problems not necessarily needing assistance from medical care, but to have a comprehensive coverage of use of hospital resources.

The characteristics of each complication and its expected impact on work capacity guided the empirical strategy for identifying complications as either an event or a chronic state in the estimation of absence from work. Most complications were considered chronic; that is, after the first hospital care contact, they were assumed to remain, with potential impact on absence from work for the remainder of the working life. This work used a dichotomous definition (yes/no) of presence of each complication and thus covered the range of impact through potential progression. Four complications (severe events of hypoglycaemia and hyperglycaemia requiring hospital care, ketoacidosis and coma) were considered events as commonly used in health economic evaluations. See also ESM Linkage of diabetes complications to costs.

Chronic state complications were: eye disease and diabetic retinopathy excluding vision loss and blindness (eye disease for short); vision loss and blindness; neuropathy; peripheral vascular disease; amputation; kidney disease; end-stage renal disease (ESRD) with dialysis or kidney transplantation; ischaemic heart disease (IHD); angina pectoris; heart failure; atrial fibrillation; diabetic foot and/or ulcers; and osteoarthritis.

Myocardial infarction and stroke allowed both event- and chronic state-related consequences. For these complications, an event was defined as an inpatient care episode including potential readmission within 30 days [14, 15]. Outpatient healthcare use with main diagnosis of myocardial infarction or stroke was considered part of the chronic state of history of the respective events.

#### Strategy for costing hospital care resource use and medications

We used diagnosis-related group (DRG) codes and the main diagnosis to assign costs to hospital-based care, pricing all NPR healthcare contacts in 2016 using contact-specific weights from the Nordic Diagnosis-Related Group (NordDRG) nomenclature [27] and the national price for a DRG weight in 2016 [28]. The proportion attributed to diabetes was estimated as the increase in costs per 100 people compared with control participants. Incremental costs of prescription medications for preventive type 2 diabetes treatment included glucose-lowering medication, and key preventive anti-hypertensive and lipid-lowering medications were estimated by comparison with control participants, but no attribution to individual complications was attempted.

#### Strategy for costs of absence from work

We used information from Försäkringskassan on (short-term) sickness and rehabilitation benefits, and (long-term) sickness and activity compensation, in 2016 [29]. Net days absent from work was derived using start and stop dates for periods with sick pay and the degree of sick leave. The cost of absence from work was valued by multiplying net days with age- and sex-specific earnings including payroll taxes [30] from labour market data at Statistics Sweden. The analysis included all individuals <66 years old irrespective of labour market status in 2016.

Diagnoses recorded at Försäkringskassan are insufficient as a source for attributing spells of sick leave in 2016 to a specific complication of diabetes. Instead, costs of days absent from work were attributed to listed diabetes complications using regression analyses, including matched control participants and adjusting for level of education (see the Statistical analyses section).

Costs of excess mortality in working ages were calculated based on the human capital method [30]. Age- and sex-specific excess mortality in the diabetes group compared with the general population were multiplied by discounted age- and sex-specific net earnings, including pay roll taxes and accounting for mean work force activity up to 65 years of age.

### Statistical analyses

#### Descriptive analyses

The descriptive analyses for diabetic and control participants report demographic and socioeconomic data as number and proportion for categorical variables and as mean and standard deviation, or median and interquartile range, for continuous variables. The univariate analysis used *t* test for differences between study groups since the central limit theorem guarantees near normality of sample means in large-sample studies like ours [31]. The prevalence rates of complications in 2016 were measured as the rate per 100,000 people to illustrate long-term burden after first onset of the complication, and were compared using standardised differences.

**Fig. 1:**
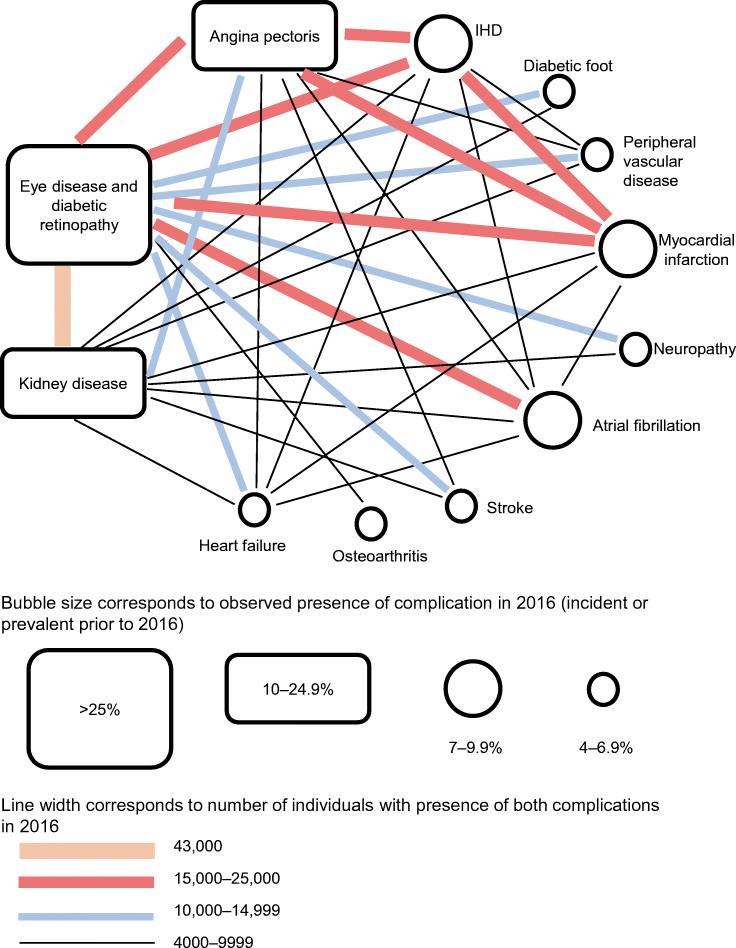
Network of comorbidity interactions for people with type 2 diabetes in 2016 (*N*=392,200)

The presence of multimorbidity was analysed using cross-tabulation of number of people with diabetes and pairwise combinations of studied diabetes complications in 2016 [8]. The results are presented as a bubble diagram where the size of the bubble indicates the proportion of people with diabetes having each complication, and the lines in between complications indicate the number of people with both complications (Fig. 1).

#### Cost analyses

The total costs of diabetes were calculated by summarising costs for hospital-based care, medication and absence from work in 2016. Ordinary least squares regression analysis investigated the correlation between costs of days absent from work and complication indicators, see Equation (1). Regression analyses have been applied in several studies of diabetes complications [6, 8, 32, 33]; in our study, the large sample size allowed the use of simple methods as the analyses depend on sample means and variances [31].

The empirical model distributed costs of days absent from work across diabetes complications, accounting for potential multiple comorbidities at the individual level, and including education as a confounding factor. It did not include demographic or other socioeconomic characteristics as the aim was to obtain an mean value across individuals irrespective of age, sex or other basic characteristics. Equation (1) shows the empirical model, including interaction effects to explore potential differences in effects of *K* complications on work absence across study groups (whereby *K* indicates the number of complications).

1 $$ Costs\ of\ days\ absent\ {from\ work}_i={\upbeta}_1+{\upbeta}_{2-4}{Education}_{i\ 2-4}+{\upbeta}_5{Diabetes}_i+{\upbeta}_{6-26}{Complications}_{i,K}+{\upbeta}_{27-42}{Diabetes}_i\times {Complications}_{i,K}+{\upvarepsilon}_i $$

for individual *i*. The estimated cost of work absence related to a complication, for an average individual with diabetes and the complication, consisted of four parts:

2 $$ {\upbeta}_{(Constant)}+{\upbeta}_{(Diabetes)}+{\upbeta}_{(Complication)}+{\upbeta}_{\left( Diabetes\times Complication\right)} $$

The first term is the regression constant β_(*Constant*)_, which is a basic mean cost of absence from work in the estimation sample. It averages costs of days absent from work for all individuals, with and without work absence, and with and without complications etc. The second term β_(*Diabetes*)_ is the basic diabetes effect, which is not attributed to any specific complication but differentiates the diabetes group from control participants, including potential differences in the distribution of other diseases not explored in this study.

Of primary interest for the analyses was the part of the costs of absence from work that was associated with complications in type 2 diabetes, which is the sum of the basic complication effect and the interaction effect β_(*Complication*)_ + β_(*Diabetes* × *Complication*)_.

## Results

### Descriptive analysis

The study population contained 392,200 people with type 2 diabetes alive and resident in Sweden in 2016, identified in health data registers since 1997 (index years median 2008), with 1,643,170 matched control participants. The mean age was 63 years in 2016, with 59% men and 41% women (Table 1). Panel attrition after the index year was mainly due to diabetes onset in individuals originally selected as control participants (5:1). It implied that while 51% of people with diabetes still had five control participants in 2016, 0.3% had no control participants and 7.7% had only one or two control participants. This attrition led to small differences in age and sex distribution between type 2 diabetes and control participants. There was generally a lower achieved level of education among people with diabetes compared with control participants (compulsory education only 30% vs 21%, university 22% vs 33%). These data confirm an expected difference in the distribution of socioeconomic characteristics between diabetic and control participants, motivating an inclusion of the level of education in regression analysis to account for these differences.

**Table 1:** Demographics of the study population

Demographic	Type 2 diabetes *N*=392,200	Control *N*=1,643,170
Index year (median; IQR)	2008 (2005; 2013)	2009 (2005; 2013)
Control participants available in 2016		
5	200,214 (51.0)	
4	106,126 (27.1)	
3	54,781 (14.0)	
2	23,202 (5.9)	
1	6849 (1.7)	
0	1028 (0.3)	
Year of birth		
<1940	39,312 (10)	123,819 (7.5)
1940–1949	139,491 (35.6)	541,711 (33)
1950–1959	109,537 (27.9)	484,661 (29.5)
1960–1969	62,681 (16)	295,487 (18)
1970–1979	25,786 (6.6)	123,517 (7.5)
1980–1989	11,007 (2.8)	52,559 (3.2)
≥1990	4386 (1.1)	21,416 (1.3)
Age in 2016, mean (SD)	62.8 (12.3)	61.5 (12.3)
Age in index year, mean (SD)	55.1 (11.5)	54.4 (11.9)
Sex		
Men	230,539 (58.8)	931,598 (56.7)
Women	161,661 (41.2)	711,572 (43.3)
Education		
Compulsory	117,989 (30.1)	351,786 (21.4)
Upper secondary	182,173 (46.5)	738,437 (44.9)
University	86,825 (22.1)	541,910 (33)
Missing	5213 (1.3)	11,037 (0.7)

Data are number (proportion) unless otherwise stated

Table 2 shows a higher morbidity among people with type 2 diabetes. The prevalence rates for ten out of 15 complications were higher in the diabetes group based on standardised differences, even though mean values were different in all complications. The largest standardised differences were found for kidney disease, eye disease and angina pectoris.

**Table 2:** Prevalence of diabetes complications in type 2 diabetic (*N* = 392,200) and control participants (*N* = 1,643,170) 2016: number of individuals with complication per 100,000 in population

Diabetes complication and concomitant conditions	Rate per 100,000 individuals		*p* value	Std diff
	Type 2 diabetes	Control		
Macrovascular complication				
IHD	9619	3288	<0.001	–0.260
Angina pectoris	10,977	3733	<0.001	–0.280
Acute myocardial infarction	8640	3058	<0.001	–0.240
Stroke	5994	2504	<0.001	–0.174
Heart failure	5118	1430	<0.001	–0.208
Atrial fibrillation	7145	4261	<0.001	–0.086
Other sudden death, cause unknown	1	0	0.0277	–0.004
Microvascular complication				
Eye disease and diabetic retinopathy^a^	43,149	28,295	<0.001	–0.337
Vision loss or blindness	374	211	<0.001	–0.039
Lower extremity disease				
Neuropathy	6167	3316	<0.001	–0.162
Peripheral vascular disease	4479	764	<0.001	–0.190
Amputation	797	34	<0.001	–0.093
Kidney disease	16,357	2260	<0.001	–0.465
ESRD^b^	1391	316	<0.001	–0.129
Other complications and events				
Hypoglycaemia	707	5	<0.001	–0.117
Hyperglycaemia	619	3	<0.001	–0.118
Ketoacidosis	143	0	<0.001	–0.061
Coma	92	13	<0.001	–0.036
Diabetic foot and ulcer	4874	1445	<0.001	–0.182
Osteoarthritis	4001	544	<0.001	–0.158

^a^Not vision loss and blindness

^b^ESRD with dialysis or kidney transplantation

Std diff, standardised difference

Figure 1 shows the diabetes-related comorbidity by describing the most prevalent diabetes complications and pairwise links of the most frequent interactions. Eye disease (43%) and kidney disease (16%) had the highest prevalence, and 43,000 individuals (11%) had both of these complications. The second most common interaction (*n* = 24,000; 6%) was between angina pectoris (11%) and eye disease. Other frequent interactions were IHD in combination with current or previous myocardial infarction (23,300; 6%), angina pectoris (22,900; 6%) and eye disease (19,800; 5%). People with myocardial infarction also had eye disease (17,400; 4%) and angina pectoris (16,300; 4%). Heart failure (*n* = 20,100; 5.1%) showed the most complexity with multiple complications: more than 4000 individuals had heart failure and at least one other of six studied complications (eye disease, kidney disease, IHD, angina pectoris, myocardial infarction and atrial fibrillation). Fewer interactions were found for the major cardiovascular complications of stroke (*n* = 23,500; 2.3%; where 5300 also had eye disease) and myocardial infarction (*n* = 33,900; 8.6%; where 8300 also had kidney disease).

### Costs of diabetes complications in 2016

People with type 2 diabetes and complications had high hospital care consumption and absence from work in 2016 (Table 3). The total costs of hospital-based care were €360 million, of which 74.7% (€269.3 million; €687 per person with diabetes) were attributed to type 2 diabetes from comparison with corresponding mean costs among control participants. Table 3 presents two alternative estimates of the total costs for days absent from work for people with complications. By the high-level analysis accounting for having at least one recorded complication event and state, respectively, absence from work amounted to €1744 million, of which 51% (€884 million; €2254 per person with diabetes) was attributed to complications (total of event and chronic state). The total costs of hospital-based care and of absence from work related to diabetes complications in 2016 were €1153 million (€2943 per person with diabetes).

**Table 3:** Diabetes complications and costs in euros in 2016: hospital-based care costs and total costs of days absent from work in 2016

Diabetes complication and concomitant conditions	Hospital-based care costs (inpatient admissions and outpatient visits) due to diabetes complications		Costs of absence from work due to diabetes complications	
	Total cost of hospital-based care (€)	Attributed to diabetes complication (%)^a^	Total costs of days absent from work (€)	Attributed to diabetes complication (%)^b^
High-level analysis—complications aggregated				
Hospital-based care (admissions and visits)	360,335,000	75		
Absence from work by regression analysis				
Event			448,026,000	21
State			1,296,035,000	61
Detailed analysis—by individual complication				
Macrovascular complications				
IHD	13,549,000	71	100,425,000	0
Angina pectoris	22,992,000	72	146,949,000	31
Acute myocardial infarction	32,248,000	68		
Event			12,588,000	11
State			102,118,000	11
Stroke	27,529,000	58		
Event			4,812,000	0
State			119,867,000	59
Heart failure	34,269,000	80	74,623,000	43
Atrial fibrillation	14,586,000	33	55,305,000	5
Other sudden death, cause unknown	1439	31		
Microvascular complications				
Eye disease and diabetic retinopathy^c^	53,187,000	57	654,577,000	16
Vision loss or blindness	108,000	59	11,558,000	48
Lower extremity disease				
Neuropathy	2,992,000	51	169,134,000	33
Peripheral vascular disease	20,383,000	87	58,233,000	30
Amputation	7,493,000	95	11,410,000	30
Kidney disease	44,526,000	85	307,539,000	24
ESRD^d^	46,531,000	89	50,858,000	51
Other complications and events				
Hypoglycaemia	5,606,000	99	15,123,000	21
Hyperglycaemia	2,379,000	100	19,605,000	38
Ketoacidosis	2,947,000	100	4,168,000	22
Coma	951,000	96	3,071,000	51
Diabetic foot and ulcer	12,052,000	91	115,173,000	36
Osteoarthritis	16,005,000	86	43,217,000	34

^a^Results from Table 5 columns (1) and (2): percentage calculated as [(1)−(2)]/(1). See also ESM Table 4

^b^Results from regression analysis in ESM Table 3 columns (1) and (2): percentage calculated as (2)/(1)

^c^Not vision loss and blindness

^d^ESRD with dialysis or kidney transplantation

The analysis of costs of absence from work by individual complications (Table 3; Detailed analysis—by individual complication) gave a higher estimate of the total costs of absence from work (€2080 million), but the costs attributed to the diabetes complications were lower (25%; €516.4 million, €1317 per person with diabetes). By this estimate, the total costs of hospital-based care and absence from work related to diabetes complications in 2016 were €785.7 million, corresponding to €2003 per person with diabetes. IHD conditions angina pectoris and myocardial infarction generated absence from work, while the regression analysis (Table 3) did not attribute additional days absent to remaining IHD conditions. A similar pattern was seen for the event of stroke, although the state of stroke caused additional absence from work indicating long-term effects. See ESM Table 3 for more details on estimations of costs related to absence from work.

Two additional cost items from the data were not distributed across complications but are included as points of reference. First, we observed 1100 additional deaths before age 66 years compared with general age-related mortality. The loss of working-age years was valued at €227 million from lost production (€579 per person). Second, insulin and other glucose-lowering medications cost €135 million in 2016. People with type 2 diabetes had 924,902 registered prescriptions of other risk-factor medications, including drugs used for the treatment or prevention of hypertension, dyslipidaemia, eye disease and neuropathy, costing a total of €45 million of which €29 million (65%) was a diabetes increment (ESM Table 4). The total added costs for diabetes related medications were €164 million (€418 per person).

Combining results in Tables 3 and 4 shows that common complications may induce high total costs, although cost per person is low. People with type 2 diabetes used more hospital care as a group: 86,104 vs 24,608 per 100,000 outpatient visits and 9894 vs 2546 per 100,000 inpatient admissions (both *p* < 0.001) (Table 4). Moreover, 26% of people with type 2 diabetes had at least one hospital contact compared with 12% for control participants (*p* < 0.001; ESM Table 5). More than half of these (15%) had ≥1 registration of eye disease, accounting altogether for 1372 inpatient admissions and 141,631 outpatient visits. The largest differences between type 2 diabetic and control participants were found among complications common in diabetes, such as kidney disease and diabetic foot and ulcers. Heart failure and kidney disease generated most inpatient admissions among people with type 2 diabetes. Atrial fibrillation had the highest rate among control participants (578 per 100,000 control participants) but this was still a lower level than in type 2 diabetes (847 per 100,000). Total costs of hospital-based care were estimated at €919 per person for the diabetes population and at €232 for the control group (Table 5 shows results per 100 people). Inpatient admissions related to complications were key drivers. Two complications also generated sizable outpatient costs: eye disease and ESRD (Table 5).

**Table 4:** Number of outpatient visits and inpatient admissions per 100,000 people for the population of type 2 diabetic (*N* = 392,200) and control participants (*N* = 1,643,170) in 2016

Diabetes complication and concomitant conditions	Outpatient visits			Inpatient admissions		
	Type 2 diabetes	Control participants	*p* value	Type 2 diabetes	Control participants	*p* value
Any event (hospital visit, admission)	86,104	24,608	<0.001	9894	2546	<0.001
Macrovascular						
IHD	2450	813	<0.001	371	99	<0.001
Angina pectoris	1183	396	<0.001	859	245	<0.001
Acute myocardial infarction	208	79	<0.001	963	316	<0.001
Stroke	327	142	<0.001	753	333	<0.001
Heart failure	2482	656	<0.001	1588	291	<0.001
Atrial fibrillation	2868	2409	<0.001	847	578	<0.001
Other sudden death, cause unknown	1	0	0.014	0	0	0.625
Microvascular						
Eye disease and diabetic retinopathy^a^	36,112	16,776	<0.001	350	151	<0.001
Vision loss or blindness	93	35	<0.001	3	1	<0.001
Lower extremity disease						
Neuropathy	1295	689	<0.001	25	8	<0.001
Peripheral vascular disease	1932	292	<0.001	623	90	<0.001
Amputation	42	1	<0.001	170	9	<0.001
Kidney disease	9381	2015	<0.001	1450	307	<0.001
ESRD^b^	22,899	183	<0.001	347	28	<0.001
Other complications and events						
Hypoglycaemia	973	5	<0.001	270	2	<0.001
Hyperglycaemia	656	3	<0.001	152	0	<0.001
Ketoacidosis	97	0	<0.001	159	0	<0.001
Coma	65	12	<0.001	47	3	<0.001
Diabetic foot and ulcer	2795	278	<0.001	412	42	<0.001
Osteoarthritis	247	42	<0.001	503	71	<0.001

^a^Not vision loss and blindness

^b^ESRD with dialysis or kidney transplantation

**Table 5:** Hospital care costs in euros per 100 people for participants with type 2 diabetes and control participants in 2016

Diabetes complication and concomitant conditions	Hospital care costs (€)			Costs of outpatient visits (€)			Costs of inpatient admissions (€)		
	(1) Type 2 diabetes	(2) Control	(3) *p* value	(4) Type 2 diabetes	(5) Control	(6) *p* value	(7) Type 2 diabetes	(8) Control	(9) *p* value
Any hospital care utilisation	91,875	23,222	<0.001	32,399	9104	<0.0001	59,477	14,118	<0.001
Macrovascular complications									
IHD	3453	982	<0.001	1045	338	<0.001	2408	644	<0.001
Angina pectoris	5860	1668	<0.001	771	264	<0.001	5090	1404	<0.001
Acute myocardial infarction	8226	2587	<0.001	95	32	<0.001	8131	2555	<0.001
Stroke	7022	2935	<0.001	148	63	<0.001	6874	2872	<0.001
Heart failure	8732	1711	<0.001	982	275	<0.001	7750	1436	<0.001
Atrial fibrillation	3717	2492	<0.001	1045	898	<0.001	2682	1594	<0.001
Other sudden death, cause unknown	<0.1	<0.1	0.749	<0.1	<0.1	0.014	<0.1	<0.1	0.625
Microvascular complications									
Eye disease and diabetic retinopathy^a^	13,558	5808	<0.001	12,270	5258	<0.001	1299	539	<0.001
Vision loss or blindness	32	11	<0.001	21	11	<0.001	11	<0.1	<0.001
Lower extremity disease									
Neuropathy	760	370	<0.001	623	338	<0.001	137	42	<0.001
Peripheral vascular disease	5195	686	<0.001	697	106	<0.001	4498	581	<0.001
Amputation	1911	106	<0.001	63	<0.1	<0.001	1848	106	<0.001
Kidney disease	11,351	1668	<0.001	3653	412	<0.001	7698	1267	<0.001
ESRD^b^	11,869	1320	<0.001	9630	1024	<0.001	2239	296	<0.001
Other complications and events									
Hypoglycaemia	1425	11	<0.001	296	<0.1	<0.001	1130	11	<0.001
Hyperglycaemia	602	<0.1	<0.001	106	<0.1	<0.001	507	<0.1	<0.001
Ketoacidosis	750	<0.1	<0.001	<0.1	<0.1	<0.001	750	<0.1	<0.001
Coma	243	11	<0.001	11	<0.1	<0.001	232	11	<0.001
Diabetic foot and ulcer	3073	285	<0.001	887	84	<0.001	2186	211	<0.001
Osteoarthritis	4076	581	<0.001	74	11	<0.001	4013	570	<0.001

^a^Not vision loss and blindness

^b^ESRD with dialysis or kidney transplantation

## Discussion

This study points to substantial costs of complications in hospital care for type 2 diabetes: the incremental costs of hospital-based care, about 75% of the total costs of complications, corresponded to €687 per person with diabetes. Costs outside the health sector were even higher in a societal perspective. With two alternative specifications of the analysis, costs of workdays absent attributed to diabetes complications were estimated at €1317 or €2254 per person, respectively. By both specifications, costs of absence from work were higher than costs of hospital-based care, indicating a significant contribution to overall societal burden of complications. Production loss related to excess mortality among people with type 2 diabetes of labour market active ages added further to total costs.

Detailed analyses of costs of complications indicated that the levels of costs of hospital-based care and absence from work for macrovascular complications were €96 million and €164 million, respectively. Microvascular complications had higher costs for hospital-based care (€136 million), and costs of absence from work were nearly twice as large as those for macrovascular complications (€287 million). The data presented underline the fact that common complications may add up to high societal costs even though the cost per person is low. Two examples of this are the observed costs for eye disease and kidney disease. Data allowed for separating of severe vision loss from earlier stages of eye disease and account for dialysis or kidney transplantation in kidney disease, but do not allow for further categorising. The estimates provide an indication of the mean value of potentially heterogeneous groups. ‘Other complications’ was a mixed group where the chronic conditions diabetic foot and ulcers and osteoarthritis were key drivers of societal costs. The results point at a remaining unmet need for risk-factor treatment as diabetes complications continue to generate substantial costs of hospital-based care and absence from work.

A further observation was that the percentage of total costs of hospital-based care attributed to type 2 diabetes and the percentage of total costs of work absence attributed to diabetes complications varied between complications (Table 3). For instance, the regression analysis did not attribute any of the costs of work absence to the IHD complication, while 71% of hospital costs were attributed to type 2 diabetes. Figure 1 shows that comorbidities were common among people with IHD. Both angina pectoris and myocardial infarctions were common in people with IHD and were associated with considerable costs of work absence attributed to the complication.

Previous estimates of the economic burden of diabetes differ from this study in objectives and design but provide relevant points of reference. A British study from 2012 estimated current and future costs of type 1 and type 2 diabetes, respectively, using aggregated data and assumptions from the literature. The results showed that diabetes accounted for 10% of total health resource expenditure in 2010/2011 in the UK, where costs of production loss exceed direct healthcare costs [5]. The investigators reported total costs and did not compare them with corresponding costs in the general population. On the other hand, the diabetes increment was estimated in the US study by the ADA from 2017, which reported that people with diabetes had 2.3 times higher medical expenditures compared with people with no diabetes [4]. This study included both type 1 and type 2 diabetes and did not report costs by individual diabetes complications. A German study from 2018 explored the economic impact of diabetes complications and their interactions using nation-wide statutory health insurance data. The results showed that an increased number of complications was associated with higher total costs [7, 8]. The German study, as well as two recent studies from Sweden [9, 10], reported total healthcare costs for people with type 2 diabetes but did not account for the fact that several complications are increasingly prevalent with age in the general population. Using the estimated costs of complications among control participants, we show that the fraction attributed to diabetes varies substantially between complications (Table 3).

The presence of diabetes complications was sourced from 20 years of individual-level longitudinal NPR data capturing complications serious enough to require outpatient physician care or an inpatient admission. Compared with studies with more limited follow-up [6, 7], this study is expected to provide a more comprehensive perspective on the long-term burden of diabetes complications. The record-linking between health data and social insurance registers for people with type 2 diabetes and their matched control participants provided a foundation for analyses of absence from work related to diabetes complications, which makes results more robust as they do not rely on recall in surveys or combination of results from many different sources as, for example, in the cited US and UK studies [4, 5]. Addressing the impact of all complications at the same time implied that our results were less vulnerable to double counting, especially considering multiple comorbidities. The study design also implied that we avoided double counting of burden of diabetes complications since we used the main diagnosis in hospital care. Still, this was a conservative approach as we might underestimate the costs since diabetes complications registered as secondary diagnosis with diabetes as main diagnosis were not included. A further strength of the analysis was that it also captured the long-term consequences of complications through data from the sickness and activity compensation scheme available for people with permanent loss of work capacity.

The study has limitations. It lacks national data on use of primary care related to diabetes complications and information about reduced productivity at work (‘presenteeism’) due to diabetes complications. Furthermore, the study design did not cover all people with diabetes but only those with diabetes indication in the working-age population. However, state of complication and use of hospital-based care were captured up to age 79 years. The Swedish National Diabetes Register (NDR), with an estimated coverage of 91.5%, reported that about 363,000 individuals with type 2 diabetes were registered in 2016 [34]. This indicates that, while the study design does not include people aged 80 years and older, the study population of 392,200 individuals still represents most of all cases of type 2 diabetes in Sweden. Moreover, the incremental costs of diabetes compared with the general population diminish with age [35]. Therefore, underestimation of the costs of complications in Sweden from onset of type 2 diabetes beyond study inclusion criteria was limited.

## Conclusion

This study shows that the burden of complications in type 2 diabetes is substantial, and costs due to absence from work are even greater than costs of hospital-based healthcare. This poses a challenge to society in terms of loss of work capacity, and more research and emphasis on guideline implementation are needed in the field of preventive treatment of type 2 diabetes complications.

## Data Availability

Subject-level data from national registers hosted by Swedish national authorities are available for research after formal evaluation of the research protocol by the Swedish Ethical Review Authority and by the respective national authorities providing data. Permission to conduct research is granted on a case-by-case basis to a limited number of named people.

## Compliance with ethical standards

ESM (PDF 295 kb)

## Abbreviations

DRG: Diagnosis-related group
ESRD: End-stage renal disease
ICD: International Classification of Diseases
IHD: Ischaemic heart disease
NBHW: National Board of Health and Welfare
NPDR: National Prescribed Drugs Register
NPR: National Patient Register
