# Supplementary material for: Costs of diabetes complications: hospital-based care and absence from work for 392,200 people with type 2 diabetes and matched control participants in Sweden
Source: Diabetologia. 2020 Sep 24;63(12):2582–94. doi: 10.1007/s00125-020-05277-3 (PMC7641955; doi:10.1007/s00125-020-05277-3)
Supplement: ESM — (PDF 295 kb) [file 125_2020_5277_MOESM1_ESM.pdf]

Electronic supplementary material (ESM)

Costs of diabetes complications: hospital-based care and absence from work for 392,200 people with type 2 diabetes and matched control participants in Sweden

Emelie Andersson; Sofie Persson; Nino Hallén; Åsa Ericsson; Desirée Thielke; Peter Lindgren; Katarina Steen Carlsson; and Johan Jendle

Table of contents

Summary of data sources 2
Population selection 4
Study group definition 6
People in labour-market active ages 6
Linkage of diabetes complications to costs 7
References 12

List of figures

ESM Fig. 1 Flowchart of population extraction from the National Board of Health and Welfare and from Statistics Sweden 4
ESM Fig. 2 Inclusion of people with diabetes; principles for handling matched controls including later transition into diabetes group if a control later meets inclusion criteria for diabetes; observation period for incidence and presence of long term complications; and observation period for resource use and costs related complications in this study. NPR – National Patient Register. NPDR – National Prescription Drug Register 5

List of tables

ESM Table 1 Summary of data sources. 2
ESM Table 2 Codes of diabetes, complications of diabetes, and concomitant disease for registry-based identification using ICD 10 codes and hospital procedure codes (KVÅ). 8
ESM Table 3 Mean and total cost of days absent from work due to diabetes complications in 2016. 9
ESM Table 4 Cost of medications in Euro used in the standard risk factor treatment in diabetes in 2016. 10
ESM Table 5 Number of individuals with ≥1 hospital visit or admission for diabetes complications or concomitant conditions per 100,000 for type 2 diabetes and controls. Corresponding total costs of hospital care for type 2 diabetes and incremental costs attributed to diabetes in Euro and percent. Data for 2016. 11

Summary of data sources

ESM Table 1 Summary of data sources.

Data source and description	Used to estimate / Analysis	+ Strengths<br>- Limitations
National Board of Health and Welfare, NBHW, a government agency under the Ministry of Health and Social Affairs. NBHW administers many national registers in the areas of healthcare and social services.<br>Presentation at: https://www.socialstyrelsen.se/en/about-us/		
National Patient Register, NPR [1]	• Identify people with type 2 diabetes<br>• Identify exposure to complications	+ Rich source of health resource use information<br>+ Main and secondary diagnoses (up to 5-digit codes) and procedure codes (5-digit codes) for hospital-based health care with national coverage<br>✓ hospital admissions (full study period)<br>✓ visits to physicians (2004-2016)<br>- No information from primary care
National Prescribed Drug Register, NPDR [2]	• Identify people with type 2 diabetes	+ Large sample<br>+ National coverage of filled prescriptions since 1 July 2005<br>- No data on medications used in hospitals or at institutions
Swedish Causes of Death Register [3]; in Swedish	• Year of death	+ Large sample<br>+ National coverage
Statistics Sweden, a government agency responsible for official statistics and for other government statistics including demographics, education, and labour market statistics.<br>Presentation at: https://www.scb.se/en/		
Register of the Total Population<br><br>Presentation at (Swedish only):<br>https://www.scb.se/vara-tjanster/bestalla-mikrodata/vilka-mikrodata-finns/individregister/registret-over-totalbefolkningen-rtb/	• Demographic data<br>• Residential region<br>• Dates for immigration and emigration	+ Large sample<br>+ National coverage

**Data source and description**	**Used to estimate / Analysis**	**+ Strengths**<br>**- Limitations**
Statistics Sweden, continued		
Register of education<br><br>Presentation at (Swedish only):<br>https://www.scb.se/vara-tjanster/bestalla-mikrodata/vilka-mikrodata-finns/individregister/registret-over-befolkningens-utbildning/	• Level of education	+ Large sample<br>+ National coverage<br>- Missing data in 1.5% (type 2 diabetes) and 0.5% (control) of the sample. Incomplete information on graduations are mainly due to foreign education where this has not been added from census information, at immigration or through targeted surveys.
Longitudinal integrated database for health insurance and labour market studies (LISA)<br>Presentation at:<br>https://www.scb.se/en/services/guidance-for-researchers-and-universities/vilka-mikrodata-finns/longitudinella-register/longitudinal-integrated-database-for-health-insurance-and-labour-market-studies-lisa/	• Socioeconomic data	+ Large sample<br>+ Database merging annual information from multiple population registers<br>+ National coverage of population ≥16 years old
Försäkringskassan (Swedish Social Insurance Agency), a government agency responsible for determining eligibility and administration of social insurance payments for sickness and functional limitations. Försäkringskassan also publish statistical reports and provide data for research.<br>Presentation at: https://www.forsakringskassan.se/omfk		
Micro Data for Analysis of Social Insurance (MiDAS) ([4]; in Swedish)	• Costs of days absent from work with sickness or rehabilitation benefits<br>• Costs of days absent from work with sickness and activity compensation	+ Large sample<br>+ National coverage of people eligible for social insurance full study period<br>+ Registers start and stop dates<br>+ Registers level of benefit and compensation enabling determination of full / partial absence<br>+ Spells ≥14 days covers full length of sick period<br>- Spells of sick leave <14 days not covered as employers are responsible for payments.

Population selection

The study population was retrieved from Swedish national health data registers according to the inclusion criteria and exclusion criteria. The National Board of Health and Welfare selected individuals from the National Patient Register, NPR, and the National Prescribed Drugs Register, NPDR, based on criteria defined by the research team. The retrospective observational design included all identified people with type 1 and type 2 diabetes in Sweden. Study subjects were in empirically observed labour market ages 16-70 years old. ESM Fig. 1 illustrates the flow chart of the population selection process. For this study, we selected from this database all people with type 2 diabetes who were alive and resident in Sweden in 2016 (n=392,200) together with their matched controls (N=1,643,170).

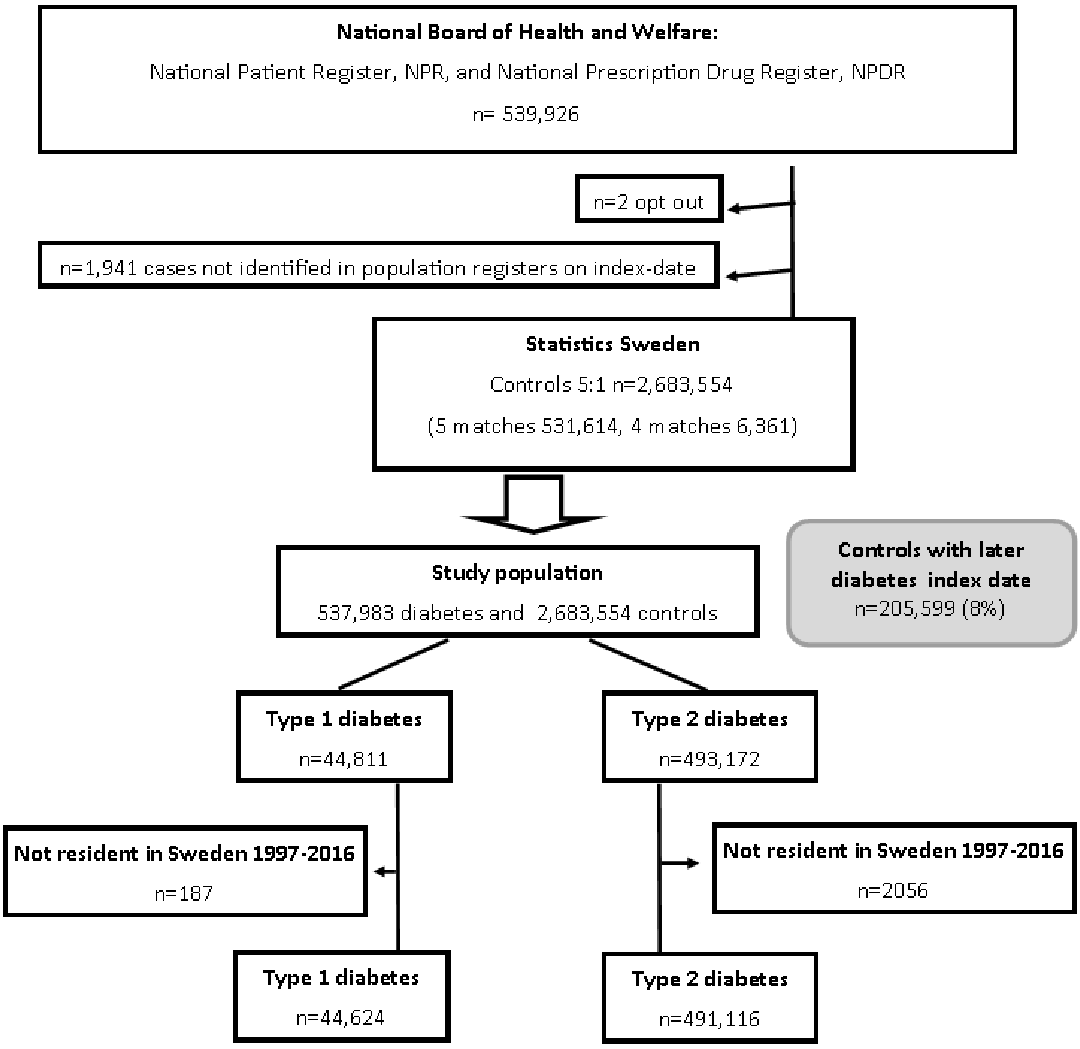

ESM Fig. 1 Flowchart of population extraction from the National Board of Health and Welfare and from Statistics Sweden.

ESM Fig. 2 presents a graphical illustration the principles for inclusion of people with diabetes, treatment of matched controls over time and measurement period for presence of complications. The illustration uses the example of one individual, Individual A, who is observed with a diabetes diagnosis in hospital-based care in 2002. At this time point, five matched controls were selected by Statistics Sweden conditioned on not being included already in the diabetes population. Of the five controls, one control meets inclusion criteria for diabetes group in 2009 and switches status from year 2009 and onwards. This individual will have his own five controls following the general principles for matched controls (not shown). In 2016 he is part of the diabetes group. One control for individual A dies in 2012 and is consequently not included in analyses of year 2016 data. In 2016, Individual A has three controls remaining in the analysis.

All costs calculations, both for health care and productivity loss, are based on data from 2016. However, our strategy to identify people with type 2 diabetes as well as the presence diabetes complications involves using information from all years available in the database (1997-2016). The costs presented in the abstract were calculated from resource use in hospitals and work absence in year 2016 only. They are all expressed as cost per person in 2016 and by the 2016 price level. It is an advantage of the study design that we can use information on onset of complications in previous years and thus in the analysis of costs of days absent from work in 2016 to allow complications to have potential long term impact. A person with a stroke in 2012 may still suffer a loss of work capacity in 2016 although he has no health care consumption stroke as main diagnosis in 2016. If we only used data on incident strokes in 2016, we would underestimate the true burden of stroke.

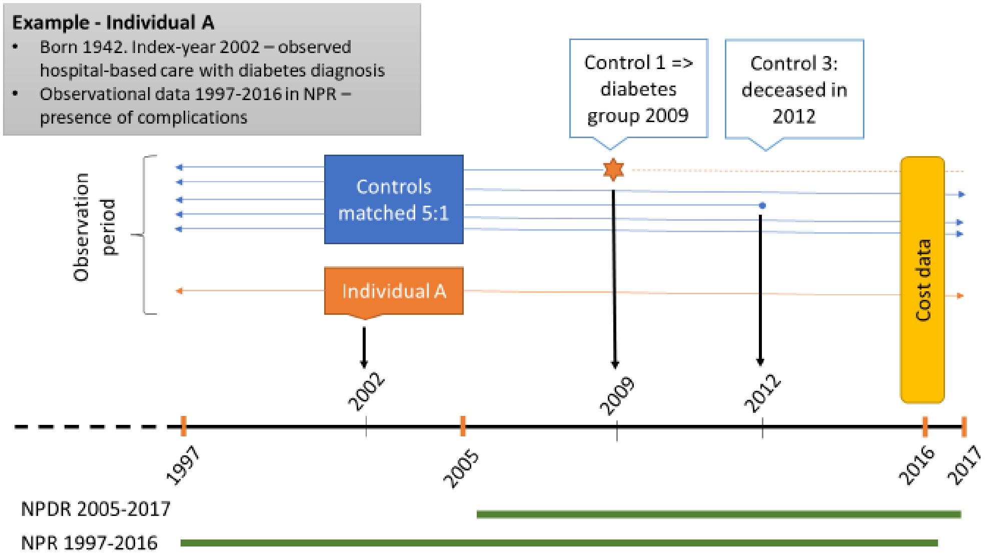

ESM Fig. 2 Inclusion of people with diabetes; principles for handling matched controls including later transition into diabetes group if a control later meets inclusion criteria for diabetes; observation period for incidence and presence of long term complications; and observation period for resource use and costs related complications in this study. NPR – National Patient Register. NPDR – National Prescription Drug Register.

Study group definition

The study used a registry-based definition of type of diabetes using available information in the NPR and the NDPR. First, we identified individuals with at least one health care contact with a registration of ICD-codes E10 (type 1 diabetes), E11 (type 2 diabetes) and E14 (unspecified diabetes) as main or supplementary diagnosis in the NPR during the full study period 1997-2017. In the register, individuals could have registrations of more than one type of diabetes. In the NPDR, we identified individuals with at least one filled prescription of insulin (ATC code A10A), and of other diabetes medications (ATC-code A10B) from Jul 2005 to Dec 2017.

We used the collated information on each individual to further sort people into either type 1 diabetes or type 2 diabetes. The division process used the following steps:

1) Clear cases of type 1 diabetes were defined as having inpatient and/or outpatient care registered with E10, but no registration of E11 or E14

    a) Subgroup with at least on prescription of insulin (A10A). This group consisted of 34,141 individuals.
    b) Subgroup with no registration in the NDPR of A10A n=10,670. About 50% of these individuals were deceased before 2005 and therefore before information on medication used was available from the NPDR

2) Clear cases of type 2 diabetes defined as having inpatient and/or outpatient care registered as E11, but no registration of E10. This group consisted of 202,501 individuals.

    c) Unclear cases of type 2 diabetes had at least one registration as E11 but also at least one registration of either E10 or E14. This group consisted of 290,671 individuals.
    d) 78% of unclear cases of type 2 diabetes had at least one registration of other diabetic drugs (A10B).

For this study, clear cases of type 1 diabetes were defined as type 1 diabetes and all other individuals as type 2 diabetes. The risk of classifying an individual with true type 1 diabetes as type 2 diabetes was considered small. Despite this, the type 2 population may contain some individuals with true type 1 diabetes, but the number was small and will be negligible with respect to impact on results.

People in labour-market active ages

The study design applied a wide perspective on labour market active ages. The database selected people meeting inclusion criteria for diabetes in ages 16-70 although many young people are still in school before age 20 years old and the flexible retirement age in Sweden allow people to retire by choice from age 61 years old.

The analysis of costs of absence from work related to diabetes complications was restricted to people <66 years old. Retirement age has become increasingly flexible over the last two decades in Sweden. The stipulated retirement age was 65 years old up to early 2000's. The right to keep a position has been extended to age 67 in recent years and is planned to be further increased to at least 69 years old. According to Statistics Sweden, the employed proportion gradually decrease with age in the general population. Merging national statistics on men and women in 2016 from the Statistical database at Statistics Sweden, 82% of 55-59-year-olds were employed and the corresponding proportion among 60-64-year-olds was 70%. Twenty seven percent of people 65-69 years old were employed in 2016.

The cut-off in the analysis of this study thus gives a conservative estimate of the total costs of work absence as it ignored data on work absence in people above 65 years old. However, given that 65 year old is still a common age of retirement and an assumption that people with health limitations are less likely to remain after this age, we found it reasonable to apply the age restriction in the present analyses of costs of absence from work in year 2016.

Linkage of diabetes complications to costs

Our strategy to identify people with type 2 diabetes as well as the presence diabetes complications in 2016 used information from all years available in the database (1997-2016). However, the costs were calculated from resource use in hospitals and work absence in year 2016 only. All costs were expressed as total cost per complication in 2016, cost per person in 2016 and derived from the 2016 price level. It is an advantage of the design that the study could use information on onset of complications in previous years in the analysis of costs of days absent from work in 2016.

The "memory" in the data allowed the analysis to account for the fact that chronic complications may have potential long-term impact on work capacity. For example, a person with a stroke in 2012 may still suffer a loss of work capacity in 2016 although she/he has no health care consumption with stroke as main diagnosis in 2016. If we only used data on incident strokes in 2016, we would underestimate the true burden of stroke. The dichotomous definition of complications (e.g. stroke yes/no) implied that the analysis did not seek to distinguish between different levels of impact of the complication. It estimated the average observed costs of work absence related to having the complication irrespective of time since onset of the chronic complication. It was beyond the scope of the study to e.g. explore whether people with repeated hospitalizations for a complication differed in work absence compared to people with no repeated hospitalizations of the same complication.

ESM Table 2 Codes of diabetes, complications of diabetes, and concomitant disease for registry-based identification using ICD 10 codes and hospital procedure codes (KVÅ).

**Diabetes complications and concomitant conditions**	**ICD 10 codes or procedural codes**	**Reference**
Type 2 or unspecified diabetes	E11, E14	
Angina pectoris	I20	[5-7]
Acute myocardial infarction	I21	[5, 6, 8]
Ischemic heart disease	I22-I25	[5, 6, 8]
Atrial fibrillation	I48	[7-9]
Heart failure	I50	[5-10]
Stroke	I61, I63, I64, I67.9	[5-7, 9]
Peripheral vascular disease, PVD	I70.2, I73.1, I73.9, I79.2, E11.5, E14.5	[5, 7]
Lower extremity amputation	KVÅ: NEQ19, NEQ99, NFQ09, NFQ19, NFQ99,<br>NGQ09, NGQ19, NGQ99, NHQ09, NHQ11, NHQ12, NHQ13, NHQ14, NHQ16, NHQ17, NHQ99	[5, 7, 10]
Other sudden death, cause unknown	R96.0, R96.1	[6, 7]
Diabetic foot and ulcers	E11.6, E14.4, L00, L03, L08, L97	[10]
Eye disease and diabetic retinopathy	H0-H4, H50-H53, H55, H57-H59, E103, E113, E143<br>KVÅ: DT006	[5]
Vision loss or blindness on one or two eyes	H54	[5]
Symptomatic neuropathy	E11.4D, G62.9, G56, G58.9, G99, G63.2, G73.3, M14.6	[10]
Kidney disease	E11.2, E14.2<br>E11.7, E11.8, E14.7, E14.8, R809, N00-N08, N10-N19, N28.9	[5, 9]
End-stage renal disease, ESRD, with dialysis or kidney transplantation	Dialysis Z49, Z94, Z99.2<br>KVÅ Dialysis DR015, DR016, DR017<br>KVÅ Transplantation KAS10, KAS20, VF420	[5, 9]
Hyperglycaemia	R73.9	[7, 11]
Ketoacidosis with/without coma	E11.0A, E11.1A, E14.1	[7, 11]
Hypoglycaemia without/with coma	E11.0C, E11.6, E11.6A, E14.0C, E14.6A, E16.0, E16.1, E16.2	[7, 11]
Coma (unspecified)	E11.0, E14.0, R40.2	[7]
Osteoarthritis	M15-M19	

Note: KVÅ - Klassifikation av vårdåtgärder [Classification of health care interventions; NBHW]

ESM Table 3 Mean and total cost of days absent from work due to diabetes complications in 2016.

**Diabetes complications and concomitant conditions**	**Costs of days absent from work in 2016: Regression analysis estimates (EUR)**			
	**(1)**<br><br>**Mean cost per person with complication**	**(2)**<br><br>**of which attributed to complication per se**	**(3)**<br><br>**Individuals with type 2 diabetes <66 years old with complication (n)**	**(4)**<br><br>**Total cost attributed to type 2 diabetes [a]**
High level analysis – complications aggregated [b]				
Event	10,918	2,305	41,037	94,597,000
State	12,968	7,899	99,941	789,394,000
Detailed analysis – by individual complication [c]				
Macrovascular complications				
Ischemic heart disease	8,549	0	11,747	0
Angina pectoris	12,495	3,812	11,761	44,834,000
Acute myocardial infarction				
Event	9,766	1,083	1,289	1,396,000
State	9,704	1,022	10,523	10,751,000
Stroke				
Event	9,704	0	712	0
State	20,992	12,310	5,710	70,289,000
Heart failure	15,245	6,562	4,895	32,122,000
Atrial fibrillation	9,178	495	6,026	2,984,000
Microvascular complications				
Eye disease and diabetic retinopathy [d]	10,308	1,625	63,503	103,209,000
Vision loss and blindness	16,824	8,142	687	5,593,000
Lower extremity disease				
Neuropathy	13,018	4,336	12,992	56,330,000
Peripheral vascular disease (PVD)	12,377	3,694	4,705	17,381,000
Amputation	12,483	3,801	914	3,474,000
Kidney disease	11,360	2,677	27,073	25,843,000
ESRD [e]	17,653	8,970	2,881	56,330,000
Other complications and events				
Hypoglycaemia	10,958	2,276	1,380	3,141,000
Hyperglycaemia	14,044	5,362	1,396	7,485,000
Ketoacidosis	11,086	2,404	376	904,000
Coma	17,547	8,865	175	1,551,000
Diabetic foot and ulcers	13,640	4,957	8,444	41,858,000
Osteoarthritis	13,200	4,517	3,274	14,790,000

[a] Rounded [b] Regression analysis estimated mean costs and proportion attributed to diabetes complication from model including covariates “any complication event” and “any complication state”. [c] Regression analysis estimated mean costs and proportion attributed to diabetes complication from model including a covariate for each complication. [d] Not vision loss and blindness. [e] End-stage renal disease with dialysis or kidney transplantation.

ESM Table 4 Cost of medications in Euro used in the standard risk factor treatment in diabetes in 2016.

**Type of medication (ATC code)**	**Type 2 diabetes (N=392,200)**			**Control (N=1,643,170)**			**Total costs for 392,200 people with type 2 diabetes (in EUR 1,000)**	**Percent attributed to type 2 diabetes**
	**Number of prescriptions**	**Medication costs (in EUR 1,000)**	**Medication cost per person (EUR)**	**Number of prescriptions**	**Medication costs (in EUR 1,000)**	**Medication cost per person (EUR)**		
Glucose lowering medications								
Insulins and analogues (A10A)	848,124	72,789	186	NA	NA	NA	72,789	100
Blood glucose lowering drugs, excl. insulins (A10B)	1,698,283	61,949	158	NA	NA	NA	61,949	100
Total costs glucose lowering medications (A10)		134,738	344	NA	NA	NA	134,738	100
Other risk factor medications								
Hypertension (C03, C07, C08, C09)	3,111,450	22,706	58	4,269,058	35,130	21	14,321	63
Dyslipidemia (C10)	1,105,568	14,146	36	1,052,175	16,429	10	10,224	72
Eye disease and diabetic retinopathy (S01LA04)	18	21	0	37	40	<0.1	11	54
Neuropathy (N06AB, N06AA09, N03AX12, N03AX16, N06AX21)	512,620	8,125	21	907,478	14,922	9	4,563	56
Total other risk factor medications		44,997	115		66 521	40	29,120	65
Total glucose lowering + other risk factor medications		314,473	458		66 521	40	163,858	91

Not applicable (NA)

ESM Table 5 Number of individuals with ≥1 hospital visit or admission for diabetes complications or concomitant conditions per 100,000 for type 2 diabetes and controls. Corresponding total costs of hospital care for type 2 diabetes and incremental costs attributed to diabetes in Euro and percent. Data for 2016.

**Diabetes complications and concomitant conditions**	**# individuals per 100,000 with ≥1 visit or admission**			**Type 2 diabetes**		
	**Type 2 diabetes**	**Control**	**p-value**	**Total costs type 2 diabetes [a]**	**Incremental costs attributed to diabetes**	**Percent**
Any event	26,461	11,630	<0.001	360,335,000	269,259,000	75
Macrovascular complications						
Ischemic heart disease	2,017	674	<0.001	13,549,000	9,683,000	71
Angina pectoris	1,229	398	<0.001	22,992,000	16,454,000	72
Acute myocardial infarction	935	318	<0.001	32,248,000	22,089,000	68
Stroke	800	351	<0.001	27,529,000	16,036,000	58
Heart failure	1,975	541	<0.001	34,269,000	27,579,000	80
Atrial fibrillation	1,872	1,385	<0.001	14,586,000	4,805,000	33
Other sudden death, cause unknown	1	0	0.028	1,000	0	31
Microvascular complications						
Eye disease and diabetic retinopathy [b]	14,817	7,501	<0.001	53,187,000	30,418,000	57
Vision loss and blindness	67	37	<0.001	108,000	64,000	59
Lower extremity disease						
Neuropathy	778	418	<0.001	2,992,000	1,525,000	51
Peripheral vascular disease (PVD)	1,087	179	<0.001	20,383,000	17,709,000	87
Amputation	175	9	<0.001	7,493,000	7,094,000	95
Kidney disease	5,185	652	<0.001	44,526,000	37,981,000	85
ESRD [c]	1,028	192	<0.001	46,531,000	41,360,000	89
Other complications and events						
Hypoglycaemia	707	5	<0.001	5,606,000	5,571,000	99
Hyperglycaemia	619	3	<0.001	2,379,000	2,373,000	100
Ketoacidosis	143	0	<0.001	2,947,000	2,947,000	100
Coma	92	13	<0.001	951,000	914,000	96
Diabetic foot and ulcers	1,240	197	<0.001	12,052,000	10,932,000	91
Osteoarthritis	594	92	<0.001	16,005,000	13,725,000	86

[a] Rounded [b] Not vision loss and blindness. [c] End-stage renal disease with dialysis or kidney transplantation.

References

[1] National Board of Health and Welfare (NBHW) (2019) The National Patient Register. Available from https://www.socialstyrelsen.se/en/statistics-and-data/registers/register-information/the-national-patient-register/. Accessed 2019 Nov 04

[2] National Board of Health and Welfare (NBHW) (2019) Läkemedelsregistret [in Swedish; National Prescribed Drugs Register]. Available from https://www.socialstyrelsen.se/statistik-och-data/register/alla-register/lakemedelsregistret/. Accessed 2019 Nov 04

[3] National Board of Health and Welfare (NBHW) (2019) Causes of Death Register. Available from https://www.socialstyrelsen.se/statistik-och-data/register/alla-register/dodsorsaksregistret/. Accessed 2019 Nov 22

[4] Försäkringskassan (the Swedish Social Insurance Agency) (2019) Micro Data for Analysis of Social Insurance (MiDAS) (in Swedish), . Available from https://www.forsakringskassan.se/statistik/kontakta-statistikenheten. Accessed 2019 November 22

[5] Adamsson Eryd S, Svensson AM, Franzén S, Eliasson B, Nilsson PM, Gudbjörnsdottir S (2017) Risk of future microvascular and macrovascular disease in people with Type 1 diabetes of very long duration: a national study with 10 ‐ year follow ‐ up. Diabetic Medicine 34(3): 411-418. https://doi.org/10.1111/dme.13266

[6] Kiadaliri AA, Gerdtham U-G, Nilsson P, Eliasson B, Gudbjörnsdottir S, Steen Carlsson K (2013) Towards Renewed Health Economic Simulation of Type 2 Diabetes: Risk Equations for First and Second Cardiovascular Events from Swedish Register Data. PLoS ONE 8(5): e62650. https://doi.org/10.1371/journal.pone.0062650

[7] Lung TW, Petrie D, Herman WH, et al. (2014) Severe Hypoglycemia and Mortality After Cardiovascular Events for Type 1 Diabetic Patients in Sweden. Diabetes Care. https://doi.org/10.2337/dc14-0405

[8] Steineck I, Cederholm J, Eliasson B, et al. (2015) Insulin pump therapy, multiple daily injections, and cardiovascular mortality in 18,168 people with type 1 diabetes: observational study. BMJ 350: h3234. https://doi.org/10.1136/bmj.h3234

[9] Tancredi M, Rosengren A, Svensson A-M, et al. (2015) Excess Mortality among Persons with Type 2 Diabetes. New England Journal of Medicine 373(18): 1720-1732. https://doi.org/10.1056/NEJMoa1504347

[10] Sørensen J, Ploug UJ (2013) The Cost of Diabetes-Related Complications: Registry-Based Analysis of Days Absent from Work. Economics Research International 2013: 1-8. https://doi.org/10.1155/2013/618039

[11] Nyström T, Bodegard J, Nathanson D, Thuresson M, Norhammar A, Eriksson JW Second line initiation of insulin compared with DPP-4 inhibitors after metformin monotherapy is associated with increased risk of all-cause mortality, cardiovascular events, and severe hypoglycemia. Diabetes Research and Clinical Practice 123: 199-208. https://doi.org/10.1016/j.diabres.2016.12.004
